# Role of the FOXM1/CMA/ER stress axis in regulating the progression of nonalcoholic steatohepatitis

**DOI:** 10.1002/ctm2.70202

**Published:** 2025-02-09

**Authors:** Shuoyi Ma, Erzhuo Xia, Miao Zhang, Yinan Hu, Siyuan Tian, Xiaohong Zheng, Bo Li, Gang Ma, Rui Su, Keshuai Sun, Qingling Fan, Fangfang Yang, Guanya Guo, Changcun Guo, Yulong Shang, Xinmin Zhou, Xia Zhou, Jingbo Wang, Ying Han

**Affiliations:** ^1^ State Key Laboratory of Cancer Biology Xijing Hospital of Digestive Diseases, The Fourth Military Medical University Xi'an China; ^2^ Department of Gastroenterology The Air Force Hospital From Eastern Theater of PLA Nanjing China; ^3^ Science and Technology Innovation Research Institute Tangdu Hospital, The Fourth Military Medical University Xi'an China

**Keywords:** chaperone‐mediated autophagy, cholesterol, endoplasmic reticulum stress, FOXM1, nonalcoholic steatohepatitis

## Abstract

**Background/aims:**

The molecular mechanisms driving nonalcoholic steatohepatitis (NASH) progression are poorly understood. This research examines the involvement of chaperone‐mediated autophagy (CMA) in NASH progression.

**Methods:**

Hepatic CMA activity was analysed in NASH mice and patients. Lysosome‐associated membrane protein 2A (LAMP2A) was knocked down or overexpressed to assess the effects of hepatocyte‐specific CMA on NASH progression. Mice received a high‐fat diet or a methionine and choline‐deficient diet to induce NASH. Palmitic acid was employed to mimic lipotoxicity‐induced hepatocyte damage in vitro. The promoter activity of FOXM1 was evaluated via ChIP and dual‐luciferase reporter assays.

**Results:**

Hepatic CMA activity was substantially low in NASH mice and patients. LAMP2A knockdown resulted in hepatocyte‐specific CMA deficiency, which promoted fibrosis and hepatic inflammation in NASH mice. Both in vitro and in vivo, CMA deficiency also exacerbated hepatocyte damage and endoplasmic reticulum (ER) stress. Mechanistically, CMA deficiency in hepatocytes increased cholesterol accumulation by blocking the degradation of 3‐hydroxy‐3‐methylglutaryl coenzyme A (HMGCR), a key cholesterol synthesis‐related enzyme, and the accumulated cholesterol subsequently induced ER stress and hepatocyte damage. The restoration of hepatocyte‐specific CMA activity effectively ameliorated diet‐induced NASH and ER stress in vivo and in vitro. FOXM1 directly bound to LAMP2A promoter and negatively regulated its transcription. The upregulation of FOXM1 expression impaired CMA and enhanced ER stress, which in turn increased FOXM1 expression, resulting in a vicious cycle and promoting NASH development.

**Conclusions:**

This study highlights the significance of the FOXM1/CMA/ER stress axis in NASH progression and proposes novel therapeutic targets for NASH.

**Key points:**

- Chaperone‐mediated autophagy (CMA) deficiency in hepatocytes promotes hepatic inflammation and fibrosis in mice with nonalcoholic steatohepatitis (NASH) by inducing cholesterol accumulation and endoplasmic reticulum (ER) stress.
- Upregulated FOXM1 impairs CMA by suppressing the transcription of lysosome‐associated membrane protein 2A (LAMP2A), a rate‐limiting component of CMA.
- ER stress increases FOXM1 expression and cholesterol accumulation.
- FOXM1/CMA/ER stress axis forms a vicious circle and promotes the development of NASH.

## Graphical abstract

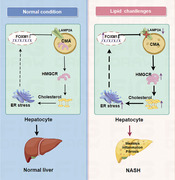

## 1 INTRODUCTION

Nonalcoholic fatty liver disease (NAFLD) has affected approximately 25% of adults globally in the span of the last 40 years, now standing as the leading chronic liver disease worldwide.^1^ Additionally, NAFLD can cause liver transplantation, primary liver cancer and end‐stage liver disease. Moreover, it stands as a major contributor to global liver‐related mortality, thus imposing substantial economic burden.^2^ The spectrum of NAFLD extends from nonalcoholic fatty liver (NAFL) and its severe form, nonalcoholic steatohepatitis (NASH) or NASH‐related cirrhosis. NASH may cause progressive liver injury in some cases, ultimately progressing to cirrhosis or hepatocellular carcinoma.^3^ Although NASH has been investigated intensively, effective treatment options are lacking at present. Therapeutic strategies for NASH, including bariatric surgery, enhanced exercise or caloric restriction, result in satisfactory outcomes only when used early.^4^

Fibrosis, inflammation and liver damage, along with steatosis are key features of NASH. Aggravated liver inflammation and fat accumulation mark the transition of NAFL to NASH. NAFLD initiation and progression are significantly influenced by excessive triglyceride accumulation in the liver.^5^ It has been demonstrated that NASH progression is significantly affected by the accumulation of cholesterol, especially nonesterified cholesterol, compared to triglycerides and fatty acids.^6^, ^7^ Excessive cholesterol accumulation in hepatocytes can launch endoplasmic reticulum (ER) stress, produce mitochondrial dysfunction and TAZ protein stabilisation, leading to cell death, inflammation and fibrosis.^8^, ^9^, ^10^ However, the molecular mechanisms underlying cholesterol accumulation in hepatocytes remain largely unclear.

Chaperone‐mediated autophagy (CMA) involves selective targeting of KFERQ motif‐containing proteins by heat shock protein family 70 for lysosomal degradation. Substrates are transported to lysosomes via the substrate–chaperone complex, which then translocate into the lysosome lumen after interacting with the lysosome‐associated membrane protein 2A (LAMP2A), a critical element of CMA. The levels of LAMP2A are directly associated with CMA activity.^11^ CMA is essential for maintaining cellular lipid metabolism.^12^ We previously demonstrated that hepatic CMA is impaired in steatosis,^13^ but its role in NASH progression is unclear.

Here, we employed hepatocyte‐specific LAMP2A‐knockout (LAMP2A^ΔHep^) mice to investigate the mechanisms underlying CMA during NASH progression. CMA deficiency led to cholesterol accumulation by impeding the degradation of 3‐hydroxy‐3‐methylglutaryl coenzyme A (HMGCR), an essential cholesterol synthesis‐related enzyme, and aggravated ER stress, thereby promoting NASH progression. In addition, FOXM1 inhibited the transcription of LAMP2A, suggesting that lipid accumulation initiated the FOXM1/CMA/ER stress feedback loop. These findings highlight that hepatic CMA is essential for NASH progression and reveal that the FOXM1/CMA/ER stress feedback loop in hepatocytes is a novel pathway contributing to NASH progression.

## 2 METHODS

### 2.1 Human sample collection

Test and control liver biopsies were obtained from biopsy‐proven NASH patients and hepatic hemangiomas patients, respectively, admitted at Xijing Hospital (ethics approval number: KY20213371‐1). There were no reports of other liver diseases, viral hepatitis, alcohol use or diabetes in the control group. Samples analysis and NAFLD activity score (NAS)^14^ were evaluated independently by two blinded pathologists. The sum of the hepatocyte ballooning scores, inflammation and steatosis constituted the NAS, and patients were diagnosed with NASH if their NAS was ≥5. Approval for this study was obtained from the Ethics Committee of the Xijing Hospital. Each patient provided informed consent prior to liver biopsy or surgery.

### 2.2 In vivo analysis

In order to generate liver‐specific LAMP2A‐conditional‐knockout mice (Albumin‐Cre‐L2A^fl/fl^; referred to as L2A^△hep^ throughout this study), mice harbouring the floxed LAMP2A allele (L2A^fl/fl^) were crossed with C57BL/6 albumin‐Cre mice. Male C57BL/6 wildtype (WT), L2A^fl/fl^ and L2A^△hep^ mice (aged 8–10 weeks) were used in subsequent experiments.

WT mice were subjected to 3‒6‐month feeding with a normal chow diet (ND) or high‐fat diet (HFD) (D12492, Research Diets) (*n* = 6–8/group) or 4‐week feeding with a diet deficient in methionine and choline (MCD) (Research Diets) (*n* = 6–8/group) to establish NASH models. L2A^fl/fl^ mice were used as WT controls. L2A^△hep^ and L2A^fl/fl^ mice were subjected to a HFD or MCD (Research Diets) for 3 months (*n* = 8–10/group) or 3 weeks, respectively (*n* = 8–10/group), to induce NASH. For LAMP2A overexpression in mice, WT mice were administered adeno‐associated virus (AAV)‐nontargeting control or AAV encoding murine LAMP2A (AAV‐LAMP2A) (2 × 10^9^ plaque‐forming units) via tail vein injection. AAVs were synthesised by Genechem.

### 2.3 In vitro analysis

HepG2 and LX2, immortalised human hepatocyte and hepatic stellate cell lines (State Key Laboratory of Cancer Biology), respectively, were grown in Dulbecco's Modified Eagle Medium (DMEM) (Gibco) containing 10% (v/v) foetal bovine serum (FBS; Gibco) and 1% penicillin–streptomycin. THP‐1 cells, a human monocytic cell line, were maintained in Roswell Park Memorial Institute(RPMI) 1640 medium (Gibco) supplemented with 10% (v/v) FBS (Gibco) and 1% penicillin–streptomycin. LAMP2A knockout (LAMP2A[‒]) was induced in HepG2 cells using the Cre‒loxP system, whereas LAMP2A overexpression was induced using a lentivirus (LV‐LAMP2A) synthesised by Genechem. FOXM1 knockdown was induced using a lentivirus from Genechem. Cell culture was conducted at 37°C and 5% (v/v) CO_2_ in a humidified incubator. Cycloheximide (CHX), palmitic acid (PA) and leupeptin (Leup) were purchased from Sigma‒Aldrich, while atorvastatin was procured from Geed Laboratory Practice Bioscience.

For the coculture experiment, 24‐h PA‐treated (300 µM) HepG2 cells or LAMP2A(‒) cells were washed and cocultured with LX2 or THP‐1 cells for 16 h in a Transwell chamber (hepatocytes and LX2 or THP‐1 cells were plated in the upper and lower chambers, respectively). For qRT‒PCR and Western blot analyses, LX2 or THP‐1 cells were harvested.

### 2.4 Primary hepatocyte isolation

Collagenase perfusion was used to isolate primary hepatocytes (PHs) from 8–10‐week‐old mice. Freshly prepared hepatocytes were suspended in DMEM containing 10% FBS and 1% penicillin–streptomycin, plated in six‐well plates (1 × 10^6^ cells/well), and rinsed with phosphate‐buffered saline (PBS) after 3 h of incubation. The medium was replaced with fresh high‐glucose DMEM supplemented with 10% FBS.

### 2.5 Dual‐luciferase reporter assay

In 24‐well plates, HepG2 cells were cultured and subjected to transfection with plasmids (pGL3‐Firefly and Renilla encoding LAMP2A) for 48 h. After harvesting and lysing the cells, their luciferase activity was evaluated by use of a dual‐luciferase reporter (DLR) assay system (Promega), as instructed by the manufacturer. Renilla luciferase activity served as the internal control. This experiment was conducted in triplicates for each plasmid construct.

### 2.6 Chromatin Immunopreci pitation (ChIP) assay

The Magna ChIP G Assay Kit (EMD Millipore) was used to perform the ChIP assay. To crosslink HepG2 cells, 1% formaldehyde was applied for 10 min at room temperature, followed by quenching with glycine. Following cell lysis, DNA was immunoprecipitated using anti‐FOXM1 antibodies (Cell Signaling Technology) for PCR amplification of FOXM1‐binding sites.

### 2.7 Histological and immunohistochemical analyses

To evaluate hepatic lipid content, liver tissue samples were frozen at an optimal cutting temperature, sectioned (thickness, 5 µm) and treated with oil red O (Sigma). For haematoxylin and eosin (H&E) and Sirius red staining, liver tissue samples were fixed in 4% formalin overnight, paraffin‐embedded, cut into 5‐µm‐thick slices, and treated with H&E or Sirius red. For immunohistochemical staining, 3% H_2_O_2_‐treated liver tissue sections (30 min) were first treated for 30 min with goat serum, followed by overnight incubation in a humidified incubator at 4°C with rabbit anti‐LAMP2A (ab125068, Abcam), rabbit anti‐FOXM1 (#20459, CST), rabbit anti‐F4/80 (ab111101, Abcam) and rabbit anti‐myeloperoxidase (MPO) (ab208670, Abcam). Subsequent to rinsing in PBS, the sections received 1‐h incubation with an IgG‐Horseradish Peroxidase secondary antibody at room temperature. We detected signals by use of a DAB kit (ZSGB‐BIO) and scanned using CaseViewer. The Image‐Pro Plus software was employed to assess the images.

### 2.8 Free cholesterol measurement

Free cholesterol (FC) in liver tissue and cells was measured by use of the Micro FC Content Assay Kit (BC 1895, Solarbio), as per the manufacturer's protocol. To summarise, liver tissues were homogenised in isopropanol, while cells were rinsed thrice with PBS before lysing in isopropanol on ice with an ultrasonic crusher. To prepare for FC measurement, the homogenates or lysates were subjected to 10‐min centrifugation (10 000 × *g*, 4°C), followed by supernatant collection.

### 2.9 8‐Hydroxyguanine immunofluorescence staining

In a fluorescent chamber, cells underwent 15‐min fixation (4% paraformaldehyde), PBS rinsing and blocking using goat serum, followed by overnight incubation with primary antibody (MA88258, AntiProtech) at 4°C and with Alexa Fluor 488‐conjugated mouse secondary antibodies (Life Technologies). The Olympus confocal microscope was used to obtain images.

### 2.10 Reactive oxygen species quantification

We determined intracellular reactive oxygen species (ROS) levels by culturing HepG2 cells in six‐well plates (2 × 10^5^ cells/well) for 24 h and treating them with 10 µM 2′,7′‐dichlorodihydrofluorescein diacetate (H_2_DCFDA; Sigma) at 37°C in the dark for 30 min. These cells were subsequently harvested, resuspended in fresh medium, and analysed via flow cytometry (FC).

### 2.11 Cell apoptosis assay

Cells were plated in a six‐well plate (1 × 10^6^ cells/well), followed by PA treatment. The adherent cells and culture supernatant were obtained and subjected to centrifugation (1500 rpm, 10 min and 4°C). Subsequently, the BD Pharmingen PE Annexin V Apoptosis Detection Kit (559763, BD Biosciences) was utilised for cell apoptosis assessment, as per the guidelines of the manufacturer. To summarise, following two washes with cold PBS, the cells were resuspended in 500 µL of binding buffer, incubated for 15 min with 5 µL of annexin V‐PE and 5 µL of 7‐AAD in the dark. In the end, the cells were treated with 300 µL of binding buffer and examined via FC.

### 2.12 Western blot assay

We prepared hepatic tissue homogenates or cell lysates in radioimmunoprecipitation buffer with protease and phosphatase inhibitors (Beyotime) for total protein extraction. The protein concentration was measured, and equal volumes were separated on an 8% sodium dodecyl sulphate–polyacrylamide gel before being transferred to a nitrocellulose membrane. The membrane was blocked using 5% milk and incubated overnight with rabbit anti‐LAMP2A (ab125068, Abcam), rabbit anti‐FOXM1 (#20459, CST), rabbit anti‐collagen I (ab260043, Abcam), mouse anti‐αSMA (A2547, Sigma), mouse anti‐β‐actin (A1987, Sigma), rabbit anti‐p‐JNK (#4668, CST), rabbit anti‐p‐EIF2S1 (ab32157, Abcam), rabbit anti‐p‐IRE1 (ab48187, Abcam) and rabbit anti‐HMGCR (ab174830, Abcam). Next day, 1‐h secondary antibody incubation was carried out at room temperature. The Image Lab system was used for visualising the protein bands.

### 2.13 Quantitative reverse transcription PCR

TRIzol (T9424, Sigma) was utilised for total RNA isolation, and the PrimeScript RT Master Mix (RR036A, Takara) was employed for reverse transcription. Quantitative PCR was implemented by use of the TB Green Premix Ex Taq II (DRR820A, Takara) in the CFX96 Touch Real‐Time PCR System (Bio‐Rad). Table S1 contains the sequences of primers for each transcript. Normalisation of target gene mRNA expression was carried out with β‐actin as a reference. This experiment was performed in triplicate.

### 2.14 Transcriptomic analysis

The RNeasy Mini Kit (Qiagen) was utilised for total RNA extraction. The Qubit 3.0 fluorometer (Life Technologies) and Nanodrop One spectrophotometer (Thermo Fisher Scientific) were employed to determine RNA concentration and quality, respectively. RNA integrity determination was achieved by use of the Agilent 2100 Bioanalyser (Agilent Technologies) utilised for, and samples with RNA integrity number >7.0 were chosen for sequencing. For paired‐end library construction, 1 µg of RNA was utilised with the mRNA‐seq Lib Prep Kit (ABclonal), according to the guidelines. Poly‐A‐containing mRNA was isolated with poly‐T oligo‐attached magnetic beads, fragmented at 94°C for 8 min by use of divalent cations, and reverse transcribed into first‐strand cDNA with random primers and reverse transcriptase. Thereafter, second‐strand cDNA synthesis was carried out by use of DNA polymerase I and RNase H. Subsequent to end repair, the addition of a single ‘A’ base, and adapter ligation, the cDNA products were purified and PCR‐amplified to generate the cDNA library. The Qubit 2.0 fluorometer (Life Technologies) quantified the purified libraries, while the Agilent 2100 bioanalyser validated the insert size and determined molar concentrations. After diluting the library to 10 pM, clusters were created using the cBot. Sinotech Genomics Co. performed library preparation and sequencing on the Illumina NovaSeq 6000 platform (Illumina). Differential expression was defined as genes with adjusted *p* < .05 and |log2 (fold change)| > 0. The raw data were uploaded to the GEO dataset (GSE274949).

### 2.15 Lipidomic analysis

Lipids were extracted from liver tissues using a solvent mixture (methyl tert‐butyl ether:methanol, 5:1) containing internal standards (ISs) and centrifuged (3000 rpm, 15 min). After centrifugation, the supernatants were allowed to dry in a vacuum concentrator. The dried residues were dissolved in a Dichloromethane:MeOH:H_2_O buffer (60:30:4.5) and subjected to centrifugation (12 000 rpm, 15 min). The final supernatant was transferred to a new glass vial for LC‒MS analysis. A SCIEX ExionLC series UHPLC system (ACQUITY UPLC HSS T3, 1.8 µm, 2.1 × 100 mm) was used for chromatographic separation, and the AB Sciex QTrap 6500+ mass spectrometer for analysing the metabolites. To investigate the metabolome, multiple reaction monitoring, profiling was adopted, treating it as a collection of functional groups. The targets were quantified using the Skyline v20.1 software. The absolute content of individual lipids was calculated relative to the ISs.

### 2.16 Coimmunoprecipitation

LAMP2A‐overexpressing cells were resuspended in IP lysis buffer (Prod #87788, Thermo Fisher Scientific) containing protease inhibitor cocktail (Millipore, 539134). The overnight incubation of the samples with an anti‐LAMP2A antibody was followed by a 4‐h incubation with Protein A/G Mix Magnetic Beads (Millipore, LSKMAGAG02) at 4°C. Subsequent to centrifugation (1000 × *g*, 3 min), the precipitates were removed by rinsing with lysis buffer five times.

### 2.17 Glucose tolerance test

For this analysis, the mice underwent a 16‐h fasting before an intraperitoneal injection with 20% glucose (2 g/kg body weight). Tail blood glucose measurements were performed prior to glucose injection or at 15, 30, 60, 90 and 120 min post‐injection.

### 2.18 Measurement of blood biochemical indices

Serum alanine aminotransferase (ALT) and cholesterol levels were examined by use of an automatic biochemistry analyser (Hitachi 7600‐120, Hitachi). Serum Transaminase (AST) was examined by Kit (BC 1565, Solarbio).

## 3 RESULTS

### 3.1 Hepatic CMA is impaired in NASH

**FIGURE 1:**
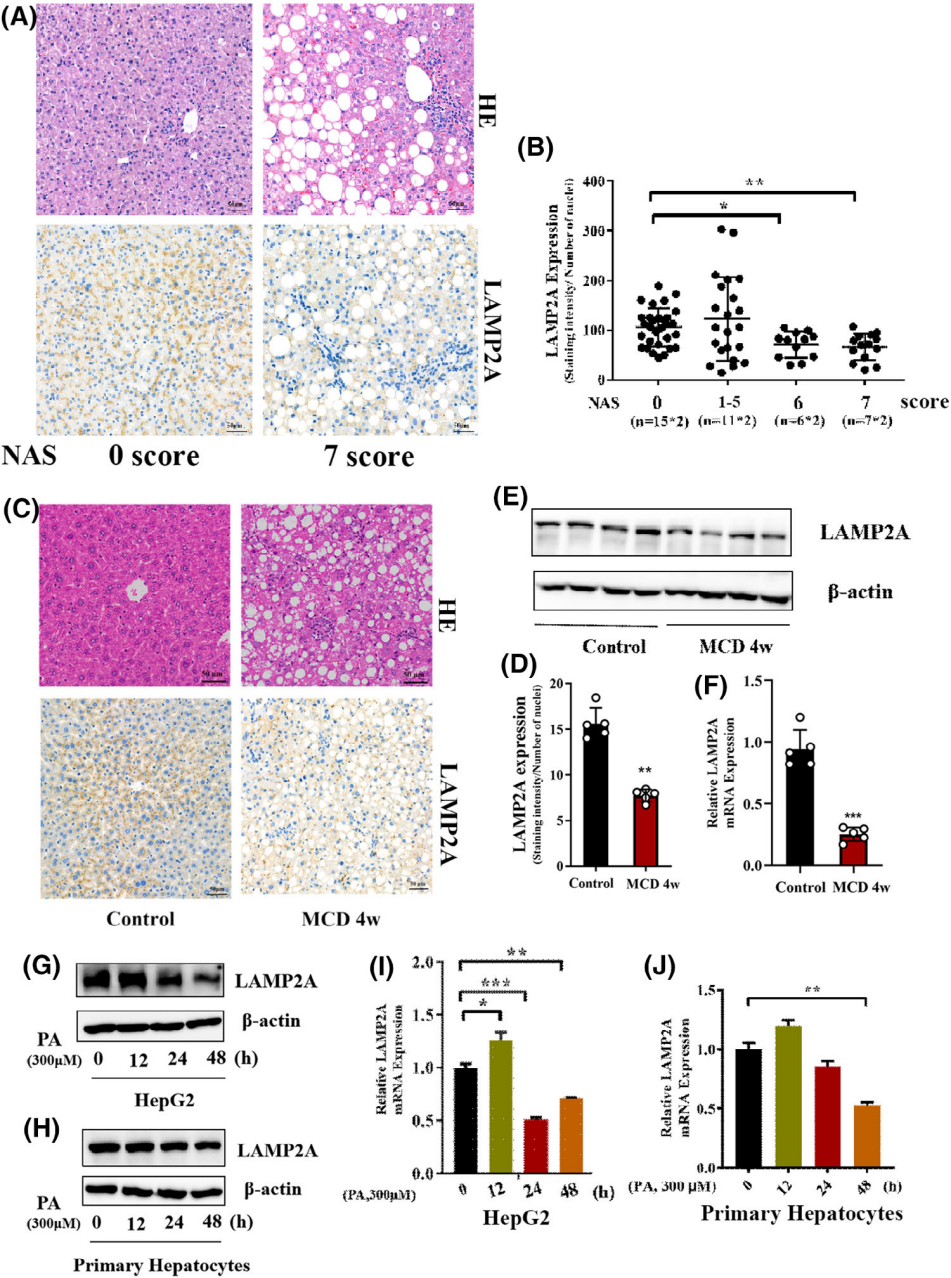
Hepatic chaperone‐mediated autophagy (CMA) is impaired in nonalcoholic steatohepatitis (NASH). (A) Representative images of haematoxylin and eosin (H&E) staining and immunohistochemical staining for lysosome‐associated membrane protein 2A (LAMP2A) in liver tissues from patients with NASH. (B) Quantitative analysis of immunohistochemical staining in patients with different NAFLD activity scores (NASs) using Image‐Pro Plus software (NAS 0, *n* = 15; 1 ≤ NAS ≤ 5, *n* = 11; NAS 6, *n* = 6; NAS 7, *n* = 7; two fields were analysed for each patient). (C) Representative images of H&E staining and immunohistochemical staining for LAMP2A in liver tissues from methionine and choline (MCD)‐fed mice. (D) Quantitative analysis of the immunohistochemical staining in (C) using Image‐Pro Plus software (*n* = 5). (E) Representative images of Western blots of LAMP2A in liver tissues from MCD diet‐fed mice. (F) qRT‒PCR results for the mRNA expression of LAMP2A in liver tissues from MCD‐fed mice (*n* = 5). (G) Western blot results for the protein expression of LAMP2A in primary hepatocytes treated with palmitic acid (PA) (300 µM). (H) Western blot results for the protein expression of LAMP2A in HepG2 cells treated with PA (300 µM). (I) qRT‒PCR results for the mRNA expression of LAMP2A in primary hepatocytes treated with PA (300 µM). (J) qRT‒PCR results for the mRNA expression of LAMP2A in HepG2 cells treated with PA (300 µM). All the data are expressed as means ± standard deviations (SDs) (^*^ *p* < .05; ^**^ *p* < .01; ^**^ *p* < .001). Significance was determined by two‐tailed unpaired Student's *t*‐test as appropriate with Prism software (GraphPad).

LAMP2A expression was evaluated in liver biopsies of NASH patients and healthy individuals to probe into the CMA's effect on NASH progression. Patients with NASH had low LAMP2A expression, with NAS scores ≥6 (Figure 1A,B). Consistently, LAMP2A protein (Figure 1C–E) and mRNA (Figure 1F) expression levels were significantly lower in the hepatic tissues of MCD‐fed NASH mice than in ND‐fed mice. To verify that CMA was impaired in hepatocytes, PHs from WT mice were isolated and exposed to vehicle or PA. Compared with the vehicle, PA markedly decreased LAMP2A protein and mRNA expression levels in PHs and HepG2 cells over time (Figure 1G–J). Altogether, these data reveal the involvement of CMA in mediating the pathological manifestations of NASH.

### 3.2 CMA deficiency in hepatocytes promotes diet‐induced NASH progression

**FIGURE 2:**
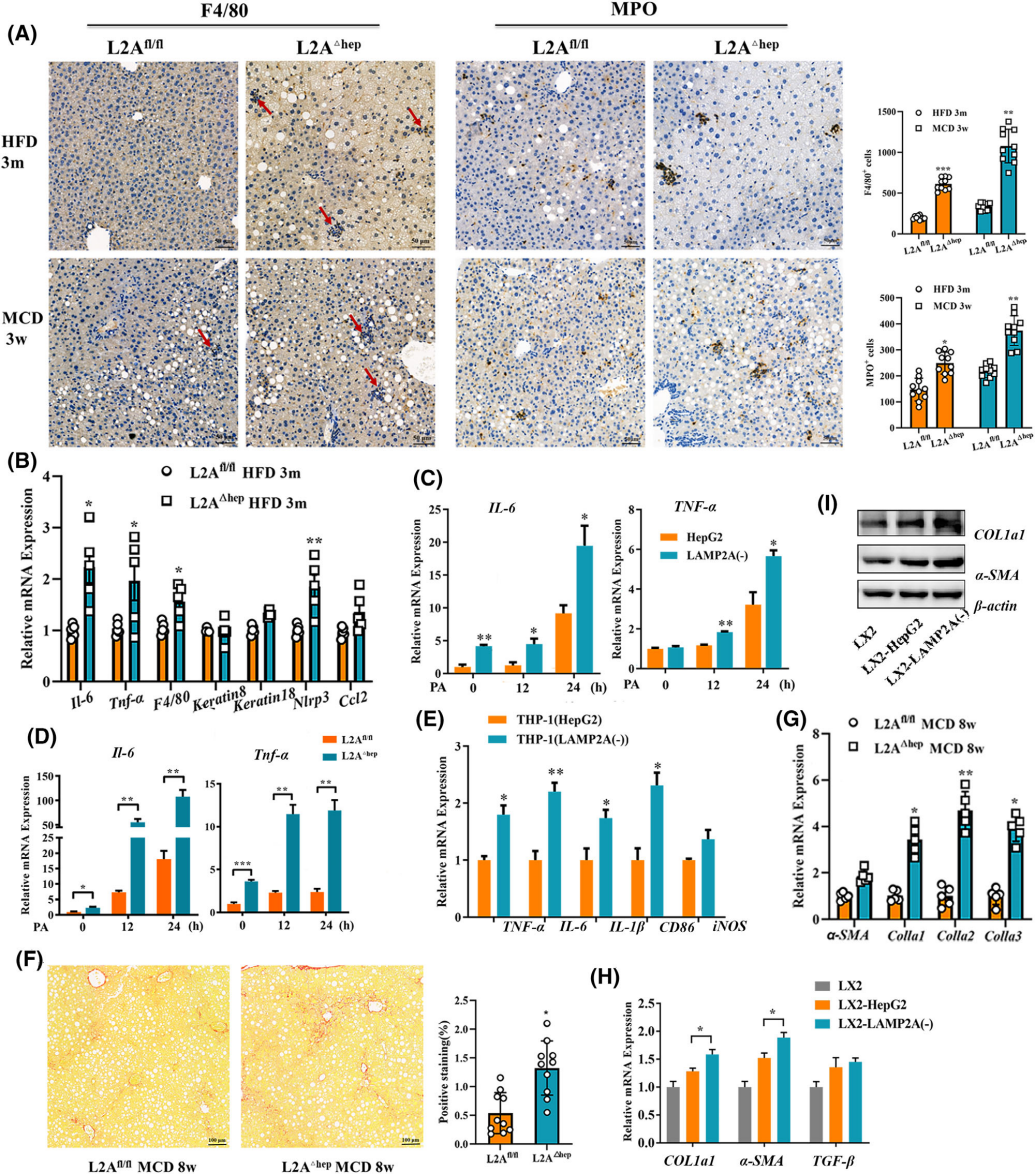
Chaperone‐mediated autophagy (CMA) deficiency in hepatocytes promotes diet‐induced hepatic inflammation and fibrosis. (A) Representative images of immunohistochemical staining for F4/80 and myeloperoxidase (MPO) (F4/80^+^ cells and MPO^+^ cells were counted using Image‐Pro Plus software). Liver tissues from LAMP2A^fl/fl^ and LAMP2A^△hep^ mice fed a high‐fat diet (HFD) for 3 months or a methionine and choline (MCD) for 3 weeks; five mice in each group and two fields in each mouse were analysed. (B) qRT‒PCR results for the mRNA expression of inflammatory cytokines in liver tissues from LAMP2A^fl/fl^ and LAMP2A^△hep^ mice fed a HFD for 3 months (*n* = 5). (C) qRT‒PCR results for the mRNA expression of interleukin‐6 (IL‐6) and tumour necrosis factor alpha (TNFα) in HepG2 and LAMP2A(‒) cells treated with palmitic acid (PA) (400 µM). (D) qRT‒PCR results for the mRNA expression of IL‐6 and TNFα in primary hepatocytes from LAMP2A^fl/fl^ and LAMP2A^△hep^ mice treated with PA (400 µM). (E) qRT‒PCR results for the mRNA expression of inflammatory cytokines in THP‐1 cells cocultured with HepG2 or LAMP2A(‒) cells treated with PA (300 µM, 24 h). (F) Representative images of Sirius red staining of liver tissues from LAMP2A^fl/fl^ and LAMP2A^△hep^ mice fed a MCD for 8 weeks (positive staining was quantified using Image‐Pro Plus software; five mice in each group and two fields in each mouse were analysed). (G) qRT‒PCR results for the mRNA expression of fibrosis‐related genes in liver tissues from LAMP2A^fl/fl^ and LAMP2A^△hep^ mice fed a MCD for 8 weeks (*n* = 5). (H) qRT‒PCR results for the mRNA expression of fibrosis‐related genes in LX2 cells cocultured with HepG2 or LAMP2A(‒) cells treated with PA (300 µM, 24 h). (I) Western blot results for the protein expression of Col1a1 and TGFβ in LX2 cells cocultured with HepG2 or LAMP2A(‒) cells treated with PA (300 µM, 24 h). All the data are expressed as means ± standard deviations (SDs) (^*^ *p* < .05; ^**^ *p* < .01; ^**^ *p* < .001). Significance was determined by two‐tailed unpaired Student's *t*‐test as appropriate with Prism software (GraphPad). LAMP2A, lysosome‐associated membrane protein 2A.

We used the Cre‒loxP system to generate hepatocyte‐specific LAMP2A‐knockout mice (LAMP2A^△hep^) and control mice (LAMP2A^fl/fl^), as reported in our previous study,^13^ to examine the function of hepatocyte CMA in NASH progression in vivo. Given that inflammation is a key feature of NASH, we probed into the impact of CMA deficiency on inflammatory responses in control and HFD‐ or MCD‐fed mice. Mice underwent 3‐month feeding with a HFD or 4‐week feeding with a MCD to induce liver inflammation. The results showed that mice with LAMP2A‐deficient hepatocytes had stronger inflammatory responses than LAMP2A^fl/fl^ mice following HFD or MCD consumption. The enhanced inflammation was evidenced by the increased infiltration of MPO^+^ neutrophils and F4/80^+^ macrophages (Figure 2A) and the markedly upregulated mRNA expression of *Il6*, *Tnfα*, *F4/80*, *Ccl2* and *Nlrp3* (Figure 2B) in liver tissue. To verify whether CMA deficiency enhances hepatocyte inflammatory responses in vitro, LAMP2A knockout was induced in HepG2 cells as reported in our previous study.^13^ Subsequently, HepG2 and LAMP2A(‒) cells were cultured in PA‐containing medium. The results revealed that proinflammatory cytokine (*IL6* and *TNFα*) expression was significantly greater in LAMP2A(‒) cells versus in control HepG2 cells after treatment with PA (Figure 2C). Similar results were observed in PHs of LAMP2A^△hep^ and LAMP2A^fl/fl^ mice (Figure 2D). After HepG2 or LAMP2A(‒) cells treated with PA were cocultured with THP‐1 cells, *IL6*, *TNFα*, *IL1β* and *CD86* expressions were significantly greater in LAMP2A(‒)‐cocultured THP‐1 cells than in the HepG2‐cocultured THP‐1 cells (Figure 2E). Fibrogenesis is another important characteristic of NASH; therefore, we examined the effects of CMA deficiency on fibrogenesis in control and MCD‐fed mice. Mice received 8‐week feeding with a MCD to provoke liver fibrogenesis. Compared to MCD‐fed LAMP2A^fl/fl^ mice, MCD‐fed LAMP2A^△hep^ mice exhibited greater collagen deposition (Figure 2F) and fibrogenesis‐related gene expression (including *αSMA*, *Col1a1*, *Col1a2* and *Col1a3*) (Figure 2G) in the hepatic tissues. After HepG2 or LAMP2A(‒) cells treated with PA were cocultured with LX2 cells in vitro, fibrogenesis‐related gene expression (*αSMA* and *COL1a1*) was greater in LX2 cells cocultured with LAMP2A(‒) cells than in LX2 cells cocultured with HepG2 cells (Figure 2H–I). Furthermore, LAMP2A^△hep^ mice exhibited more severe hepatic steatosis (Figure S1A) and higher liver‐to‐body weight ratios (Figure S1B), ALT levels (Figure S1C,E) and AST levels (Figure S1D,F) than LAMP2A^fl/fl^ control mice did, which align with the findings of our previous study.^13^ However, the two groups exhibited similar weight (Figure S1G) and glucose tolerance (Figure S1H). LAMP2A deficiency also increased lipid accumulation in both HepG2 cells (Figure S1I) and PHs (Figure S1J) in vitro. Altogether, our results indicate that reduced CMA activity is linked to severe pathological manifestations of NASH, suggesting that CMA plays a protective role in NASH.

### 3.3 CMA deficiency in hepatocytes exacerbates diet‐induced ER stress and hepatocyte damage

**FIGURE 3:**
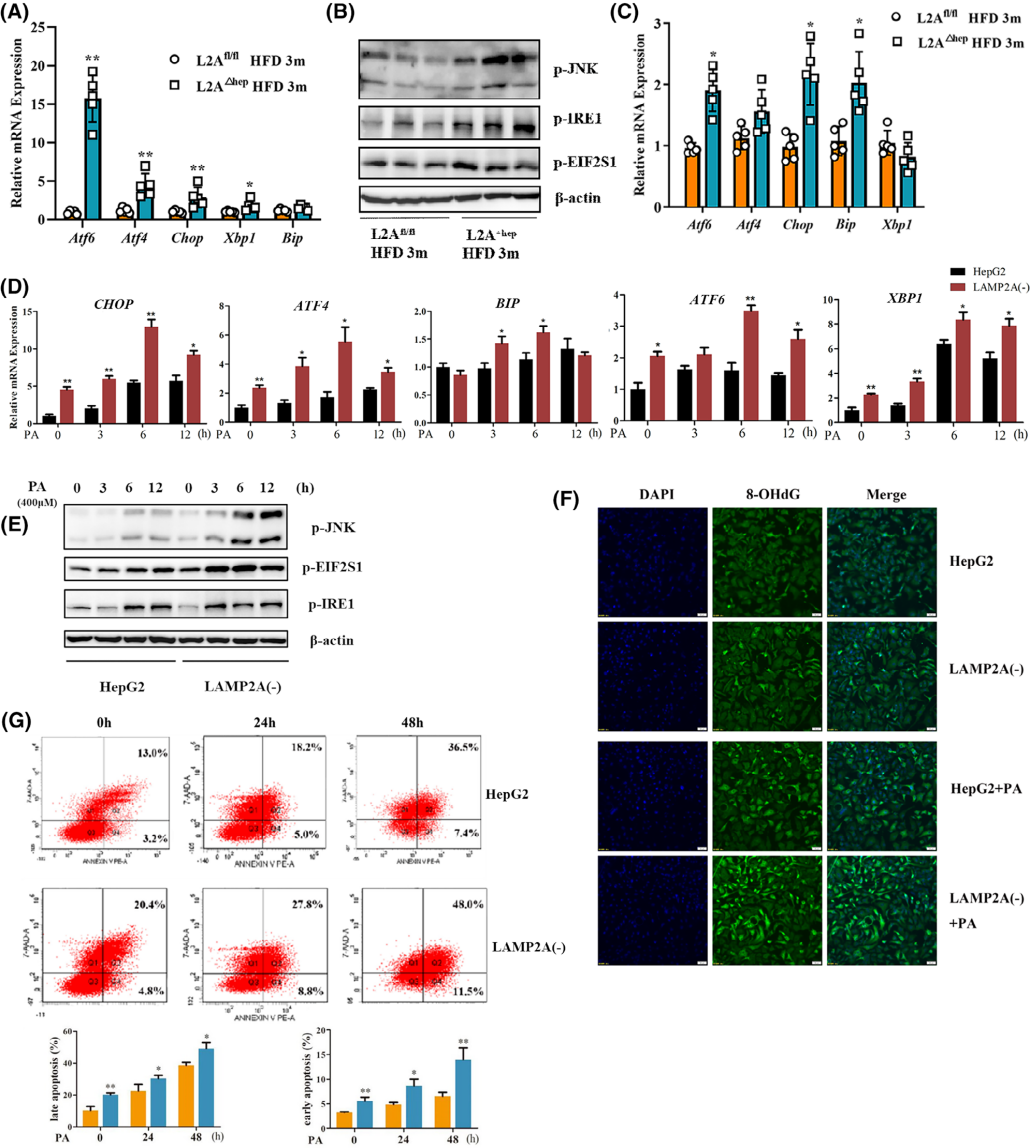
Chaperone‐mediated autophagy (CMA) deficiency in hepatocytes exacerbates diet‐induced endoplasmic reticulum (ER) stress and hepatocyte damage. (A) qRT‒PCR results of the mRNA expression of ER stress‐related genes in liver tissues from LAMP2A^fl/fl^ and LAMP2A^△hep^ mice fed a high‐fat diet (HFD) for 3 months (*n* = 5). (B) Western blot results for the protein expression of ER stress markers in liver tissues from LAMP2A^fl/fl^ and LAMP2A^△hep^ mice fed a HFD for 3 months (*n* = 3). (C) qRT‒PCR results for the mRNA expression of ER stress‐related genes in primary hepatocytes from LAMP2A^fl/fl^ and LAMP2A^△hep^ mice fed a HFD for 3 months (*n* = 5). (D) qRT‒PCR results for the mRNA expression of ER stress‐related genes in HepG2 and LAMP2A(‒) cells treated with palmitic acid (PA) (400 µM). (E) Western blot results for the protein expression of ER stress markers in HepG2 and LAMP2A(‒) cells treated with PA (400 µM). (F) Representative images of 8‐hydroxyguanosine (8‐OHdG) immunofluorescence staining of HepG2 and LAMP2A(‒) cells treated with or without PA (400 µM). (G) Flow cytometry results for the apoptosis of HepG2 and LAMP2A(‒) cells treated with PA (400 µM) and statistical analysis of early and late apoptosis. All the data are expressed as means ± standard deviations (SDs) (^*^ *p* < .05; ^**^ *p* < .01). Significance was determined by two‐tailed unpaired Student's *t*‐test as appropriate with Prism software (GraphPad). LAMP2A, lysosome‐associated membrane protein 2A.

The liver is crucial for regulating glucose and lipid homeostasis^15^, ^16^; consequently, it is more prone to ER stress, which, when prolonged, provokes cell damage or death, induce inflammatory responses and accelerate the progression of NASH.^17^ Because the abovementioned results revealed that CMA deficiency accelerated NASH progression, we examined whether CMA deficiency affects hepatic ER stress. The hepatic mRNA expression levels of transcription factors related to ER stress (including *Atf6*, *Atf4*, *Chop*, *Xbp1* and *Bip*) were significantly greater in the LAMP2A^△hep^ mice relative to LAMP2A^fl/fl^ mice after 3 months of HFD consumption (Figure 3A). The phosphorylation status of JNK, IRE1α and EIF2S1 in the cytoplasm serves as an crucial indicator of ER stress. The JNK, IRE1α and EIF2S1 phosphorylation levels were significantly greater in the hepatic tissues of LAMP2A^△hep^ mice relative to LAMP2A^fl/fl^ mice after 3 months of HFD consumption (Figure 3B). To examine the ER stress status in hepatocytes, PHs were isolated from LAMP2A^△hep^ and LAMP2A^fl/fl^ mice after 3 months of HFD consumption. ER stress was more severe in LAMP2A‐deficient hepatocytes than in control hepatocytes (Figure 3C), which is consistent with the aforementioned results. To validate the detrimental effects of CMA deficiency on ER stress in vitro, LAMP2A(‒) and HepG2 cells were subjected to PA treatment. Our findings suggested that PA treatment notably increased the transcription of ER stress‐related transcription factors (*ATF6*, *ATF4*, *CHOP*, *XBP1* and *BIP*) (Figure 3D) and the phosphorylation levels of JNK, IRE1α and EIF2S1 (Figure 3E) in LAMP2A(‒) cells over time. To examine the influence of increased ER stress on hepatocytes, DNA damage in PA‐treated LAMP2A(‒) and HepG2 cells was assessed via 8‐hydroxyguanosine (8‐OHdG) immunofluorescence staining (Figure 3F), and apoptosis was examined through annexin V staining via FC (Figure 3G). The results showed that CMA deficiency induced DNA damage and promoted apoptosis in hepatocytes in response to PA, indicating that CMA deficiency accelerates the progression of NASH by exacerbating diet‐induced ER stress and hepatocyte damage.

### 3.4 CMA deficiency in hepatocytes increases cholesterol accumulation by blocking HMGCR protein degradation

**FIGURE 4:**
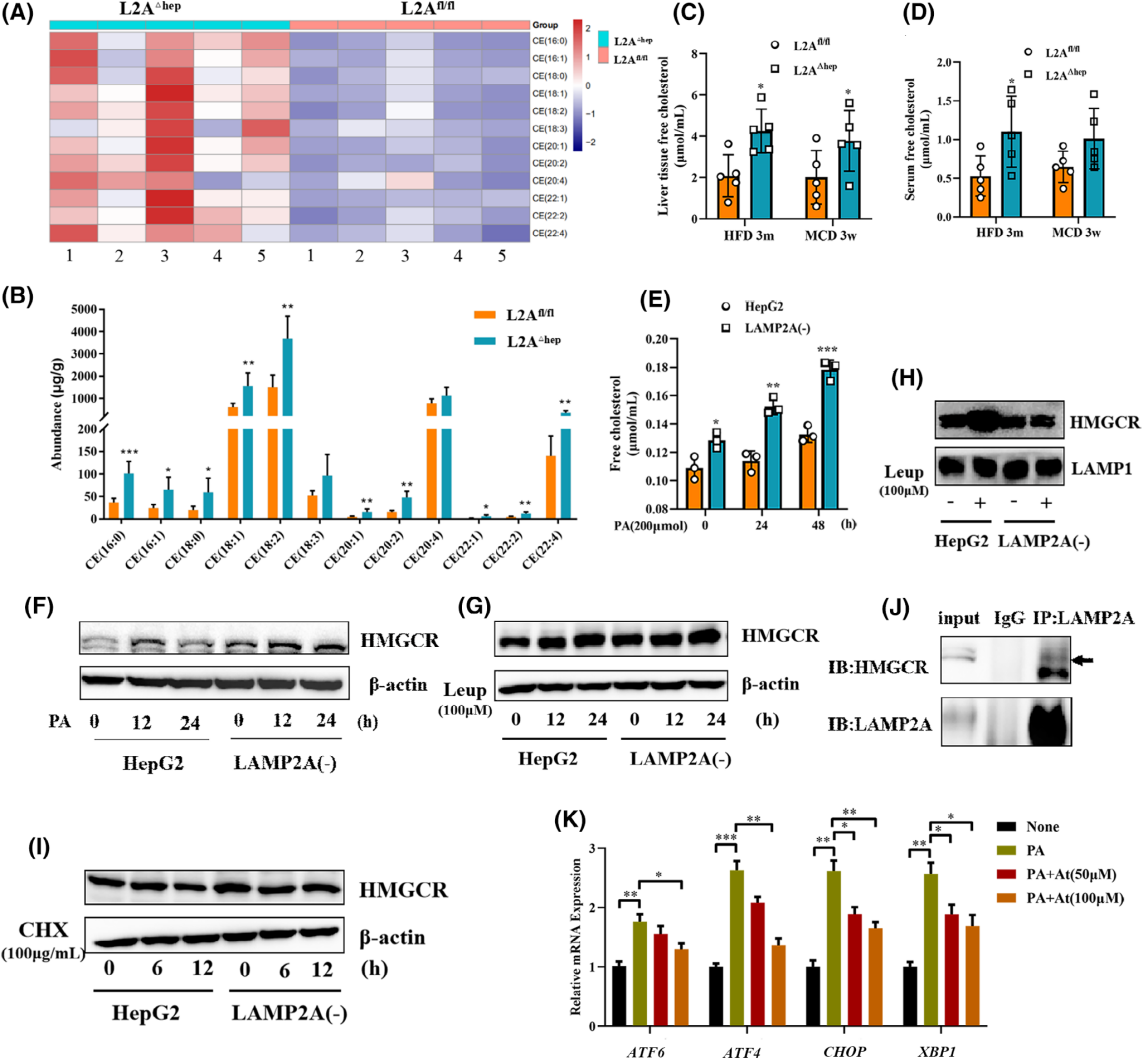
Chaperone‐mediated autophagy (CMA) deficiency in hepatocytes increases cholesterol accumulation by blocking 3‐hydroxy‐3‐methylglutaryl coenzyme A (HMGCR) protein degradation. (A) Heatmap of differentially abundant cholesterol species according to *p*‐values. (B) Relative abundance of different cholesterol species in the liver. Levels of free cholesterol in liver tissue (C) and (D) in serum from LAMP2A^fl/fl^ and LAMP2A^△hep^ mice fed a high‐fat diet (HFD) for 3 months or a methionine and choline (MCD) diet for 3 weeks. (E) Levels of free cholesterol in HepG2 and LAMP2A(‒) cells treated with palmitic acid (PA) (200 µM). (F) Western blot results for the protein expression of HMGCR in HepG2 and LAMP2A(‒) cells treated with PA (300 µM). (G) Western blot results for the protein expression of HMGCR in HepG2 and LAMP2A(‒) cells treated with leupeptin (Leup) (100 µM). (H) Western blot results for the protein expression of HMGCR in lysosomes from HepG2 and LAMP2A(‒) cells treated with or without Leup (100 µM) for 24 h. (I) Western blot results for the protein expression of HMGCR in HepG2 and LAMP2A(‒) cells treated with cycloheximide (CHX) (100 µg/mL). (J) Coimmunoprecipitation assay of HMGCR and lysosome‐associated membrane protein 2A (LAMP2A) in HepG2 cells treated with PA (300 µM) for 12 h. (K) qRT‒PCR results for the mRNA expression of endoplasmic reticulum (ER) stress‐related genes in LAMP2A(‒) cells treated with or without atorvastatin for 12 h and with PA (400 µM) for 6 h. Significance was determined by two‐tailed unpaired Student's *t*‐test as appropriate with Prism software (GraphPad).

Excessive lipids accumulating in the liver provoke hepatocyte damage and inflammation, which contribute to the pathogenesis of NASH.^18^, ^19^ Cholesterol accumulation has emerged as a major determinant of hepatic lipotoxicity.^20^ The ER regulates intracellular lipid and cholesterol content levels, and ER stress may be induced by the disturbance in lipid homeostasis.^21^ To examine the impact of CMA deficiency on hepatic lipid metabolism, the liver tissues of HFD‐fed mice were subjected to targeted lipidomic analysis. The results revealed that CMA deficiency altered the levels of various lipids (Figure S2), including free fatty acids, triacylglycerol, diglycerides and cholesterol esters (CEs); in particular, CEs were the most significantly altered lipid class. The levels of 12 CE species (including 16:0, 16:1, 18:0, 18:1, 18:2, 18:3, 20:1, 20:2, 20:4, 22:1, 22:2 and 22:4) were markedly greater in LAMP2A^△hep^ mice than in LAMP2A^fl/fl^ mice (Figure 4A,B). Because CEs are produced mainly by the esterification of FC and the lipotoxicity of FCs is greater than that of CEs, we evaluated FC levels in mouse serum and liver tissues. The FC levels were significantly higher in both the liver tissues (Figure 4C) and serum (Figure 4D) of CMA‐deficient mice relative to control HFD‐fed or MCD‐fed mice. Similarly, the levels of FCs were significantly greater in CMA‐deficient cells than in HepG2 cells treated with or without PA (Figure 4E). The increase in cholesterol may reflect an increase in de novo cholesterol synthesis; therefore, the expression of HMGCR, a key cholesterol synthesis enzyme, was assessed in vitro. Both CMA deficiency (Figure 4F) and treatment with the lysosome inhibitor leupeptin (Leup) (Figure 4G) increased the protein level of HMGCR in HepG2 cells. However, treatment with Leup did not significantly increase HMGCR protein levels in CMA‐deficient cells (Figure 4G). Furthermore, the protein expression of HMGCR in lysosomes was assessed (Figure 4H). Consistently, HMGCR protein expression was significantly elevated in lysosomes from HepG2 cells treated with Leup for 24 h, but treatment with Leup did not increase HMGCR protein expression in lysosomes from CMA‐deficient cells. These results suggest that CMA deficiency blocks the HMGCR protein from entering lysosomes. Subsequently, CHX, an inhibitor of protein synthesis, was used to examine the impact of CMA on HMGCR degradation. As depicted in Figure 4I, CMA deficiency significantly delayed HMGCR protein degradation. In addition, a coimmunoprecipitation assay validated the interaction between HMGCR and LAMP2A (Figure 4J). These results indicate that the HMGCR protein is a potential substrate for CMA. Therefore, we speculated that the CMA‐induced disruption of HMGCR degradation is responsible for hepatic cholesterol accumulation in NASH. Treatment with atorvastatin, an HMGCR inhibitor, alleviated PA‐induced ER stress in LAMP2A(‒) cells (Figure 4K). Taken together, these results demonstrate that CMA deficiency in hepatocytes leads to cholesterol accumulation by blocking HMGCR protein degradation, consequently aggravating ER stress.

### 3.5 Upregulated FOXM1 expression impairs CMA and ER stress contributes to the upregulation of FOXM1 expression in NASH

**FIGURE 5:**
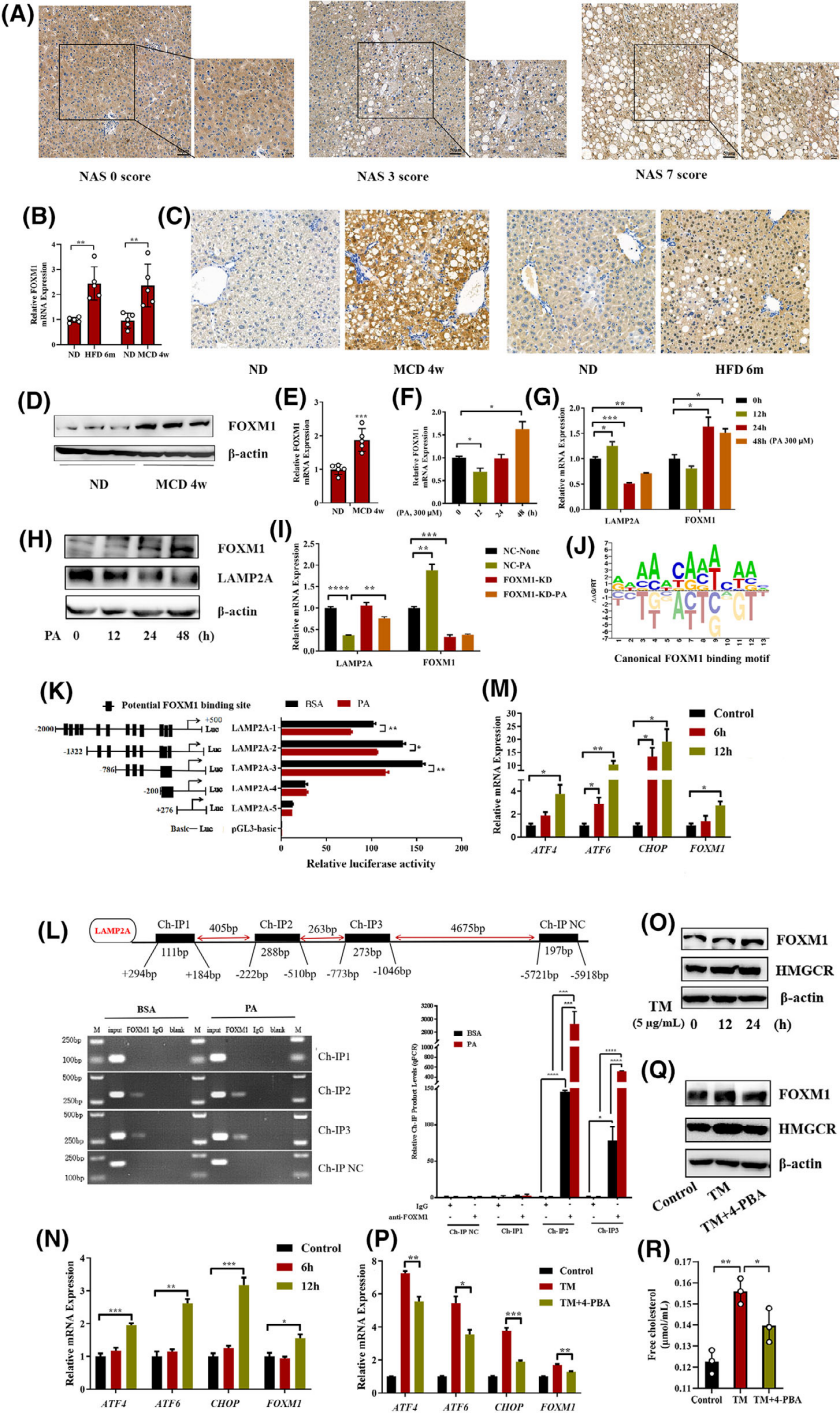
Upregulated FOXM1 expression impairs chaperone‐mediated autophagy (CMA) and endoplasmic reticulum (ER) stress contributes to upregulated FOXM1 expression in nonalcoholic steatohepatitis (NASH). (A) Representative images of immunohistochemical staining for FOXM1 in liver tissues from patients with NASH (the sample size is the same as that in Figure 1). (B) qRT‒PCR results for the mRNA expression of FOXM1 in liver tissues from high‐fat diet (HFD)‐fed or methionine and choline (MCD)‐fed mice (*n* = 5). (C) Representative images of immunohistochemical staining for FOXM1 in liver tissues from HFD‐ or MCD‐fed mice (*n* = 5). (D) Representative Western blot images of FOXM1 expression in liver tissues from mice fed MCD. (E) qRT‒PCR results for the mRNA expression of FOXM1 in primary hepatocytes from mice fed a MCD (*n* = 5). (F) qRT‒PCR results for the mRNA expression of FOXM1 in primary hepatocytes treated with palmitic acid (PA) (300 µM). (G) qRT‒PCR results for the mRNA expression of FOXM1 and lysosome‐associated membrane protein 2A (LAMP2A) in HepG2 cells treated with PA (300 µM). (H) Western blot results for the protein expression of LAMP2A and FOXM1 in HepG2 cells treated with PA (300 µM). (I) qRT‒PCR results for the mRNA expression of FOXM1 and LAMP2A in negative control cells and FOXM1‐knockdown cells treated with or without PA (300 µM). (J) Canonical FOXM1‐binding motif identified using the JASPAR database. (K) Serially truncated LAMP2A promoter fragments were cloned and inserted into pGL3‐luciferase reporter plasmids and transfected into HepG2 cells. The cells were subsequently treated with PA (200 µM) for 24 h, and the relative luciferase activity was subsequently evaluated 72 h after the end of PA treatment. (L) ChIP assays demonstrated the direct binding of FOXM1 to the LAMP2A promoter region in HepG2 cells. M, marker. qRT‒PCR analysis of the ChIP products validated the binding between FOXM1 and the LAMP2A promoter. (M) qRT‒PCR results for the mRNA expression of ER stress‐related genes and FOXM1 in HepG2 cells treated with tunicamycin (5 µg/mL). (N) qRT‒PCR results for the mRNA expression of ER stress‐related genes and FOXM1 in HepG2 cells treated with β‐mercaptoethanol (.55 mM). (O) Western blot results for the protein expression of FOXM1 and 3‐hydroxy‐3‐methylglutaryl coenzyme A (HMGCR) in HepG2 cells treated with tunicamycin (5 µg/mL). (P) qRT‒PCR results for the mRNA expression of ER stress‐related genes in HepG2 cells treated with tunicamycin (5 µg/mL) for 12 h only or then with 4‐phenylbutyric acid (4‐PBA) (1 mM) for 4 h. (Q) Western blot results for the protein expression of FOXM1 and HMGCR in HepG2 cells treated with tunicamycin (5 µg/mL) for 24 h only or then with 4‐PBA (1 mM) for 12 h. (R) Levels of free cholesterol in HepG2 cells treated with tunicamycin (5 µg/mL) for 24 h only or then with 4‐PBA (1 mM) for 12 h. All data are expressed as means ± standard deviations (SDs) (^*^ *p* < .05; ^**^ *p* < .01; ^**^ *p* < .001). Significance was determined by two‐tailed unpaired Student's *t*‐test as appropriate with Prism software (GraphPad).

Given that in NASH the mRNA expression of LAMP2A is low in hepatocytes, we examined the transcriptional regulation of LAMP2A. Transcriptomic analysis revealed 246 differentially expressed transcription factors (Figure S3A) between liver tissues from MCD‐fed mice and those from ND‐fed mice. These transcription factors intersected with 555 transcription factors of LAMP2A predicted using the Animal TF3.0 database, resulting in the identification of 88 overlapping transcription factors (Figure S3B). Among the top 10 overlapping transcription factors (Figure S3C), FOXM1 was found to be linked to hepatic inflammation and fibrosis.^22^, ^23^ Therefore, we evaluated FOXM1 expression in human liver biopsy samples. The nuclear expression of FOXM1 was higher in NASH livers compared to NAFL or normal livers, and FOXM1 was hardly expressed in normal livers (Figure 5A). Consistently, the hepatic mRNA (Figure 5B) and protein (Figure 5C,D) levels of FOXM1 were dramatically greater in the MCD‐fed mice versus ND‐fed mice. To analyse the FOXM1 expression in hepatocytes, PHs were obtained from MCD‐fed and ND‐fed mice. Similarly, FOXM1 expression was significantly greater in hepatocytes isolated from MCD‐fed mice versus those isolated from ND‐fed mice (Figure 5E). Furthermore, in vitro experiments demonstrated that PA treatment significantly increased FOXM1 expression in PHs (Figure 5F) and HepG2 cells (Figure 5G,H). In addition, FOXM1 knockdown partially counteracted the PA‐induced downregulation of LAMP2A expression (Figure 5I), suggesting a negative correlation between FOXM1 and LAMP2A expression. To determine whether FOXM1 inhibited LAMP2A expression at the transcription level, the potential binding sites of FOXM1 in the LAMP2A promoter region were predicted using the JASPAR database and validated in HepG2 cells via a DLR and ChIP assays. Our findings suggested that the PA‐induced downregulation of LAMP2A expression was regulated by potential FOXM1‐binding sites situated between −2000 and −200 bp (Figure 5K). Consistently, ChIP confirmed that FOXM1 could bind to two regions in the LAMP2A promoter (from −1046 to −773 bp and from −510 to −222 bp) (Figure 5L). These results indicated that FOXM1 suppressed LAMP2A expression by binding to its promoter in PA‐treated HepG2 cells.

Furthermore, we explored the cause of upregulated hepatic FOXM1 expression in NASH. The induction of ER stress via activators such as tunicamycin (TM) (Figure 5M,O) and β‐mercaptoethanol (Figure 5N) increased FOXM1 expression, whereas its inhibition via 4‐phenylbutyric acid (4‐PBA) (Figure 5P,Q) decreased FOXM1 expression, suggesting that ER stress may regulate FOXM1 expression. Moreover, the protein expression of HMGCR (Figure 5O,Q) and the levels of FC (Figure 5R) also changed with increasing ER stress.

Altogether, our findings suggest that upregulated expression of FOXM1 impairs CMA by suppressing the transcription of LAMP2A; CMA impairment enhances ER stress, which in turn increases FOXM1 expression and FC levels, resulting in a vicious cycle and promoting the development of NASH.

### 3.6 Increased hepatic CMA activity ameliorates diet‐induced NASH phenotype and ER stress

**FIGURE 6:**
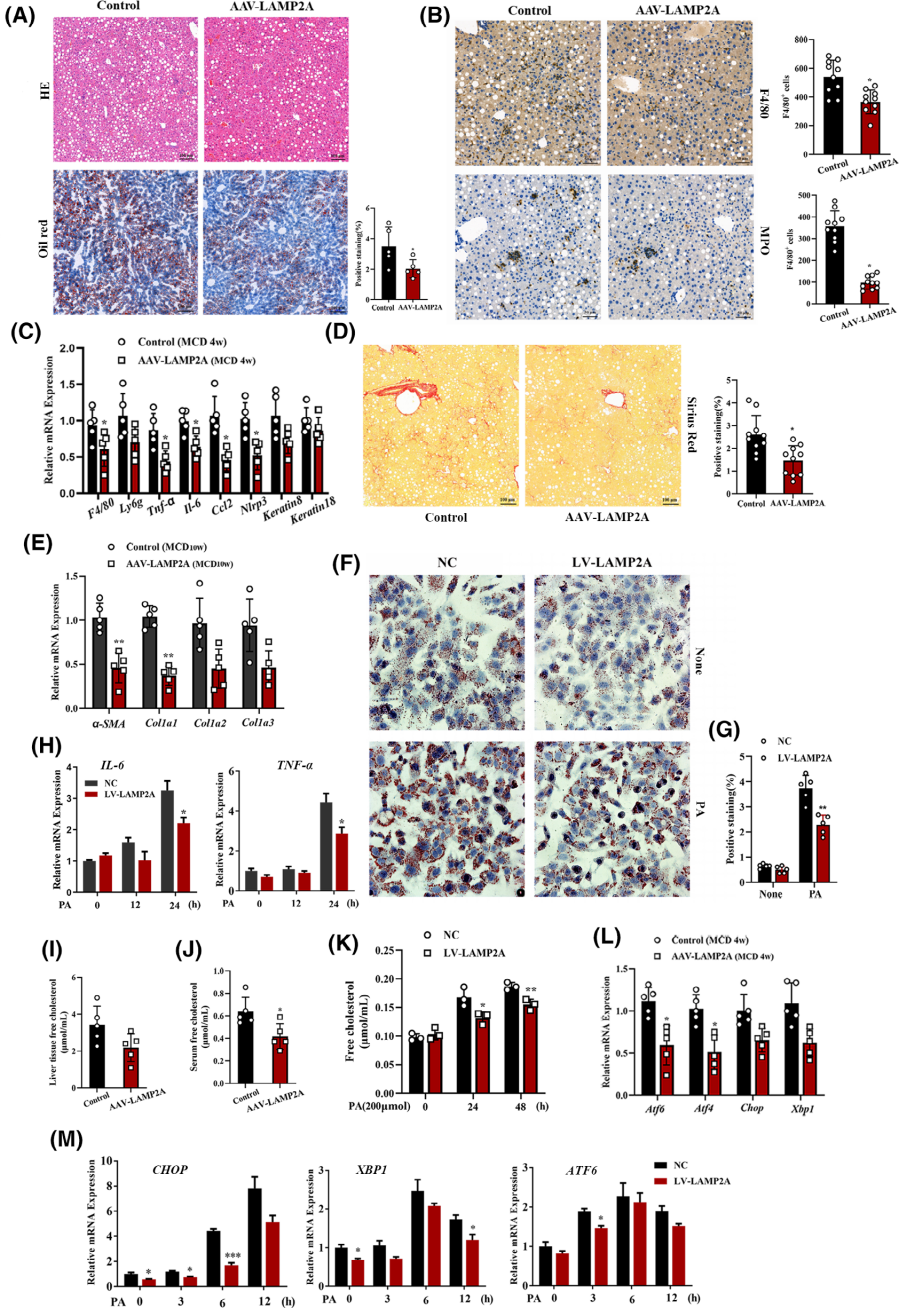
Increasing chaperone‐mediated autophagy (CMA) activity in the liver ameliorates the diet‐induced nonalcoholic steatohepatitis (NASH) phenotype and endoplasmic reticulum (ER) stress. (A) Representative images of haematoxylin and eosin (H&E) and oil red O staining of liver tissues from control and AAV‐LAMP2A mice fed a methionine and choline (MCD) diet for 4 weeks (*n* = 5) and relative quantification of oil red O staining (using Image‐Pro Plus software). (B) Representative images of immunohistochemical staining for F4/80 and myeloperoxidase (MPO) in liver tissues from control and AAV‐LAMP2A mice fed a MCD for 4 weeks and count of F4/80^+^ cells and MPO^+^ cells (F4/80^+^ cells and MPO^+^ cells were counted using the Image‐Pro Plus software; five mice in each group and two fields in each mouse were analysed). (C) qRT‒PCR results for the mRNA expression of inflammatory cytokines in control and AAV‐LAMP2A mice fed a MCD for 4 weeks (*n* = 5). (D) Representative images of Sirius red staining of liver tissues from control and AAV‐LAMP2A mice fed a MCD for 8 weeks (*n* = 5) and relative quantification of Sirius red staining (using Image‐Pro Plus software, two fields were analysed for each mouse). (E) qRT‒PCR results for the mRNA expression of fibrosis‐related genes in liver tissues from control and AAV‐LAMP2A mice fed a MCD for 8 weeks (*n* = 5). (F) Representative images of oil red O staining of negative control (NC) and LAMP2A‐overexpressing (LV‐LAMP2A) cells treated with palmitic acid (PA) (300 µM, 24 h). (G) Relative quantification of oil red O staining in (F) (using Image‐Pro Plus software, five fields from each group were analysed). (H) qRT‒PCR results for the mRNA expression of interleukin‐6 (IL‐6) and tumour necrosis factor alpha (TNFα) in NC and LV‐LAMP2A cells treated with PA (400 µM). Levels of free cholesterol in the liver tissue (I) and serum (J) of mice fed a MCD for 4 weeks (*n* = 5). (K) Levels of free cholesterol in NC and LV‐LAMP2A cells treated with PA (300 µM, 24 h). (L) qRT‒PCR results for the mRNA expression of ER stress‐related genes in control and AAV‐LAMP2A mice fed a MCD for 4 weeks (*n* = 5). (M) qRT‒PCR results for the mRNA expression of ER stress‐related genes in NC and LV‐LAMP2A cells treated with PA (400 µM). All the data are expressed as means ± standard deviations (SDs) (^*^ *p* < .05; ^**^ *p* < .01). Significance was determined by two‐tailed unpaired Student's *t*‐test as appropriate with Prism software (GraphPad). AAV, adeno‐associated virus; LAMP2A, lysosome‐associated membrane protein 2A.

We successfully induced liver‐specific LAMP2A overexpression in mice via AAV 8 (AAV‐LAMP2A) to validate the involvement of hepatocyte CMA in NASH pathogenesis (Figure S4A). Oil Red O and H&E staining suggested that the severity of hepatic steatosis significantly weakened in LAMP2A‐overexpressing mice reactive to control mice after 4 weeks of MCD consumption (Figure 6A). In addition, histological analysis revealed that LAMP2A‐overexpressing mice presented weaker inflammatory responses, lower infiltration levels of F4/80^+^ macrophages and MPO^+^ neutrophils, and lower mRNA levels of proinflammatory cytokines (*Il6*, *Tnfα*, *F4/80*, *Ccl2* and *Nlrp3*) (Figure 6B–C). Sirius red staining revealed that the fibrotic area (Figure 6D) and fibrosis‐related gene expression (*αSMA*, *Col1a1*, *Col1a2* and *Col1a3*) (Figure 6E) were lower in LAMP2A‐overexpressing mice compared to control mice after 8 weeks of MCD consumption. These results were partially validated in PA‐treated HepG2 and LAMP2A‐overexpressing cells (LV‐LAMP2A) (Figure S4B), suggesting that LAMP2A overexpression alleviated PA‐induced steatosis (Figure 6F,G) and inflammation (Figure 6H) in vitro. Furthermore, FC levels were evaluated in LAMP2A‐overexpressing MCD‐fed mice and in LAMP2A‐overexpressing cells treated with PA. The results revealed that LAMP2A overexpression in hepatocytes decreased the level of FC both in vivo (Figure 6I,J) and in vitro (Figure 6K). Finally, the ER stress status was also assessed in these mice and cells. The results revealed that the LAMP2A overexpression‐induced increase in CMA activity partially alleviated PA‐induced ER stress in vivo (Figure 6L) and in vitro (Figure 6M). Altogether, our findings suggest that increasing CMA activity in the liver effectively alleviates diet‐induced NASH and ER stress.

## 4 DISCUSSION

This study uncovered that the hepatic tissues of NASH mice and patients had markedly lower CMA activity. CMA deficiency induced by hepatocyte‐specific LAMP2A knockout aggravated liver damage, inflammation and fibrosis, in diet‐induced NASH mice. Although CMA deficiency significantly aggravated liver fibrosis in diet‐induced NASH mice, we did not observe an association between CMA activity and fibrosis levels in NASH patients. We speculate that our sample size was limited, the stage of fibrosis was early, and no patients experienced decompensation events, which may have influenced our observation of the correlation between CMA activity and fibrosis stage. To better confirm the association between CMA activity and fibrosis stage, we will further expand the sample size and include patients with different stages of fibrosis.

Lipotoxicity‐induced inflammation can trigger and aggravate hepatocyte damage, causing NASH.^24^ Recent studies have shown that NASH‐related lipotoxicity is attributed to FC accumulation. Within the ER, cholesterol synthesis is strictly regulated by HMGCR, the rate‐limiting enzyme in cholesterol biosynthesis.^25^ The disruption of cholesterol homeostasis induces ER stress, whereas sustained ER stress contributes to NASH progression. We found that CMA deficiency in hepatocytes increased cholesterol accumulation by blocking HMGCR degradation and increasing ER stress in mice with diet‐induced NASH. In contrast, enhancing CMA activity in mice and cell lines through genetic manipulation alleviated the severity of NASH. FOXM1 expression was elevated in the livers of diet‐induced NASH mice and NASH patients and inhibited LAMP2A expression at the transcription level. Decreased CMA activity aggravated ER stress, which in turn upregulated FOXM1 expression and FC levels, forming a vicious cycle and promoting NASH development.

Currently, NAFLD affects an estimated 32.4% of the global population, and its prevalence is projected to increase further.^26^ Approximately one‐third of NAFLD patients progress to inflammatory steatohepatitis and fibrosis, potentially leading to decompensated liver disease, cirrhosis and death.^27^ Therefore, it is essential to block NAFLD progression to NASH. In this study, CMA activity dramatically declined in liver tissues of NASH patients as well as NASH mice after 4‐week MCD consumption or 6‐month HFD consumption; however, it was slightly greater in liver tissues of simple steatosis patients (Figure 1A) and mice with HFD‐induced simple steatosis (3 months of HFD consumption) (Figure S5) than in the corresponding control liver tissues. Therefore, we speculate that CMA is a defense mechanism activated in hepatocytes in response to lipid accumulation in the early stage; however, it fails to protect the liver against sustained lipid accumulation. This study revealed that increasing CMA activity is a potential therapeutic strategy for NASH and may be more effective if implemented in the early stage.

Given CMA's protective function in NASH progression, we investigated how CMA deficiency affects NASH development. ER stress can induce NASH onset and progression. In particular, mild ER stress alleviates the pathological manifestations of NASH, whereas severe and sustained ER stress exacerbates the pathological manifestations by inducing inflammation, cell death and extracellular vesicle secretion.^28^ Here, we showed that mice deficient in hepatocyte‐specific CMA were more susceptible to diet‐induced NASH as it aggravates ER stress and liver damage. Lipidomic analysis revealed that CMA deficiency led to cholesterol accumulation, which induced ER stress. Consistently, a previous study reported that total LAMP2A knockout markedly increased the levels of total circulating cholesterol, whereas CMA activation markedly decreased cholesterol levels in mice.^29^ Hepatic CMA deficiency results in metabolic dysfunction owing to the failure of the timely degradation of lipid and glucose metabolism‐related proteins. In this study, HMGCR, a rate‐limiting enzyme of cholesterol biosynthesis, was identified as a potential substrate for CMA, and the abnormal degradation of HMGCR induced by CMA deficiency was found to be mainly responsible for cholesterol accumulation in NASH. Therefore, we speculate that CMA deficiency in hepatocytes promotes the development of NASH by enhancing ER stress through the disruption of cholesterol metabolism. We previously reported that CMA deficiency‐induced abnormal Nup85 degradation aggravates macrophage recruitment and promotes liver inflammation.^30^ Stellate and sinusoidal endothelial cells are essential for NASH development,^31^ but the function of CMA in these cells remains unclear. We will explore this topic in future studies.

Finally, we examined the cause of downregulated LAMP2A expression in NASH. The FOX protein family comprises transcription factors distinguished by a winged‐helix structure within the DNA‐binding domain. Among these, FOXM1 directly or indirectly regulates cell differentiation,^32^ proliferation,^33^ metabolism,^34^, ^35^ apoptosis^36^ and the maintenance of stem cell pluripotency.^37^ Additionally, it contributes to the progression and recovery of various diseases, including pulmonary fibrosis,^38^, ^39^ pneumonia,^40^, ^41^ diabetes,^42^ liver injury repair,^43^ adrenal lesions,^44^ vascular diseases,^45^ brain diseases,^46^ arthritis,^47^, ^48^ myasthenia gravis^49^ and psoriasis.^50^, ^51^ While FOXM1 has been extensively researched in various human diseases, its role in NASH warrants further investigation. This study revealed that hepatic FOXM1 expression was significantly high NASH patients and mice and was negatively correlated with LAMP2A expression. FOXM1 knockdown prevented the PA‐induced downregulation of LAMP2A expression in vitro. In addition, FOXM1 inhibited the transcription of LAMP2A. On the basis of these findings, we attempted to examine the cause of upregulated hepatic FOXM1 expression in NASH. In addition to lipid challenge, we found that ER stress could induce the upregulation of FOXM1 expression. Therefore, we speculate that the disruption of the FOXM1/CMA/ER stress axis promotes NASH progression. However, the complex role of FOXM1 in NASH warrants in‐depth investigation.

In conclusion, this study revealed that decreased CMA activity in hepatocytes leads to cholesterol accumulation and enhances ER stress, consequently accelerating NASH progression. In addition, upregulated FOXM1 expression was responsible for the decrease of CMA activity. Therefore, targeting the FOXM1/CMA/ER stress signaling cascade may represent a potential therapeutic strategy for NASH.

## AUTHOR CONTRIBUTIONS

Jingbo Wang and Ying Han supervised the project. Shuoyi Ma, Erzhuo Xia and Xia Zhou designed the experiments and wrote the manuscript. Xia Zhou, Yulong Shang and Changcun Guo participated in reviewing and editing the manuscript. Miao Zhang, Yinan Hu, Siyuan Tian and Bo Li performed the mouse studies and experiments in cells. Rui Su, Gang Ma and Keshuai Sun assisted with the animal experiments. Guanya Guo, Fangfang Yang and Qingling Fan analysed the data.

## CONFLICT OF INTEREST STATEMENT

The authors declare they have no conflicts of interest.

## Supporting information



Supporting Information
