# Supplementary material for: Role of the FOXM1/CMA/ER stress axis in regulating the progression of nonalcoholic steatohepatitis
Source: Clin Transl Med. 2025 Feb 9;15(2):e70202. doi: 10.1002/ctm2.70202 (PMC11807764; doi:10.1002/ctm2.70202)
Supplement: Supplementary file 1 — Supporting Information [file CTM2-15-e70202-s001.docx]

**Supplementary Data**


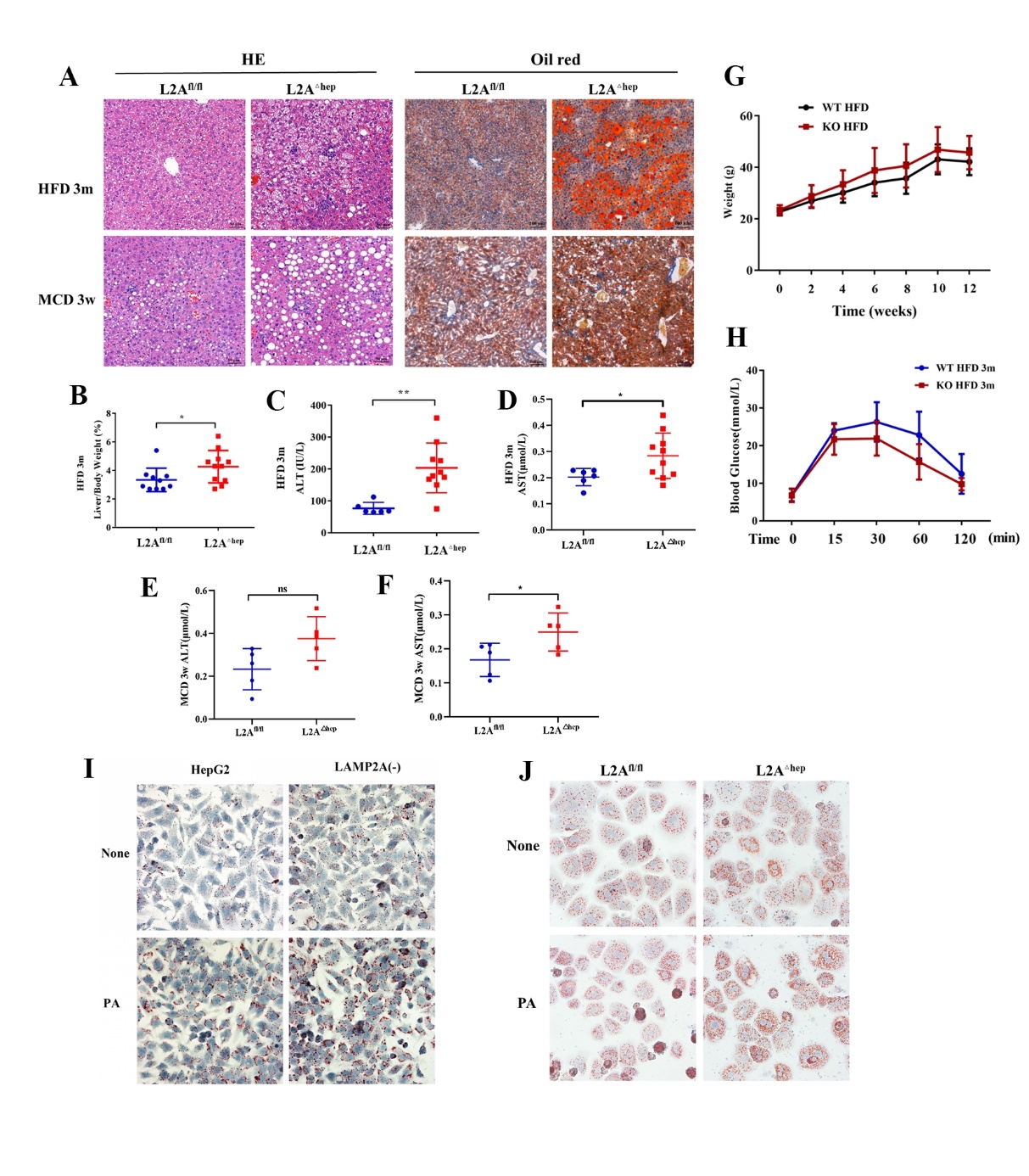


Supplementary Figure 1. **CMA deficiency in hepatocytes aggravates diet-induced hepatic steatosis and liver damage.** (A) Representative images of H&E staining and oil red O staining of liver tissues from LAMP2A^fl/fl^ and LAMP2A^△hep^ mice fed a HFD or MCD (n = 5). (B) Liver-to-body weight ratios of LAMP2A^fl/fl^ and LAMP2A^△hep^ mice fed a HFD. (C) ALT levels in LAMP2A^fl/fl^ and LAMP2A^△hep^ mice fed a HFD. (D) AST levels in LAMP2A^fl/fl^ and LAMP2A^△hep^ mice fed a HFD. (E) ALT levels in LAMP2A^fl/fl^ and LAMP2A^△hep^ mice fed a MCD. (F) AST levels in LAMP2A^fl/fl^ and LAMP2A^△hep^ mice fed a MCD. (G) Weights of LAMP2A^fl/fl^ and LAMP2A^△hep^ mice fed a HFD. (H) Glucose tolerance test of LAMP2A^fl/fl^ and LAMP2A^△hep^ mice fed a HFD. (I) Representative images of oil red O-stained HepG2 and LAMP2A(-) cells treated with PA (300 μM, 24 h) (400×). (J) Representative images of oil red O-stained primary hepatocytes from LAMP2A^fl/fl^ and LAMP2A^△hep^ mice treated with PA (300 μM, 24 h) (400×). All the data are expressed as means ± SDs (*, *P* < 0.05; **, *P* < 0.01; ns, not significant). Significance was determined by two-tailed unpaired Student’s t test as appropriate with Prism software (GraphPad).


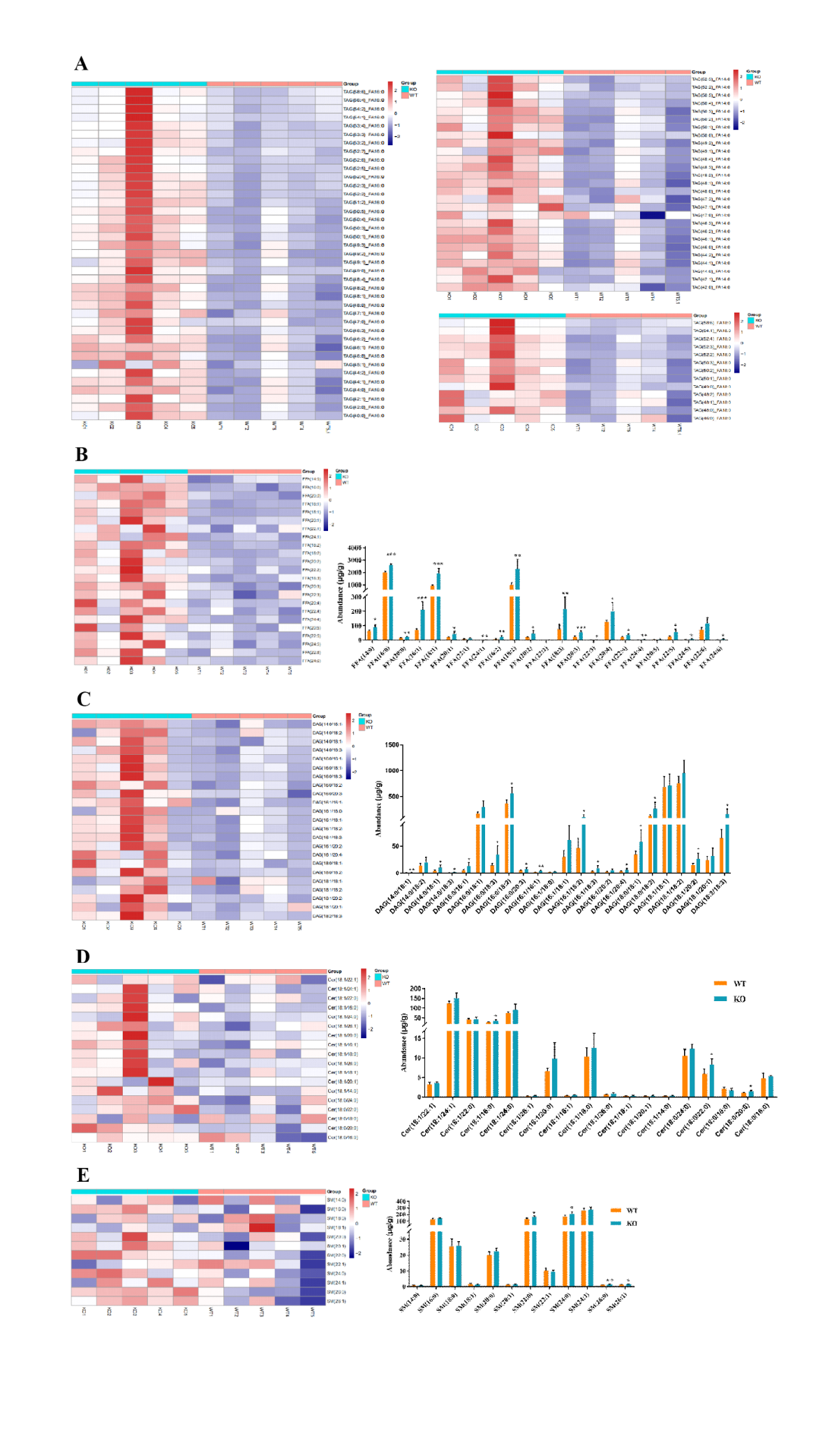


Supplementary Figure 2. **Targeted lipidomic analysis of liver tissues from HFD-fed mice.** (A) Heatmap of differentially abundant TAG species according to P values. (B) Heatmap of differentially abundant FFA species according to P values and their relative abundance in the liver. (C) Heatmap of differentially abundant DAG species according to P values and their relative abundance in the liver. (D) Heatmap of differentially abundant ceramide species according to P values and their relative abundance in the liver. (E) Heatmap of differentially abundant sphingomyelin species according to P values and their relative abundance in the liver. All the data are expressed as means ± SDs (*, *P* < 0.05; **, *P* < 0.01; ***, *P* < 0.001). Significance was determined by two-tailed unpaired Student’s t test as appropriate with Prism software (GraphPad).


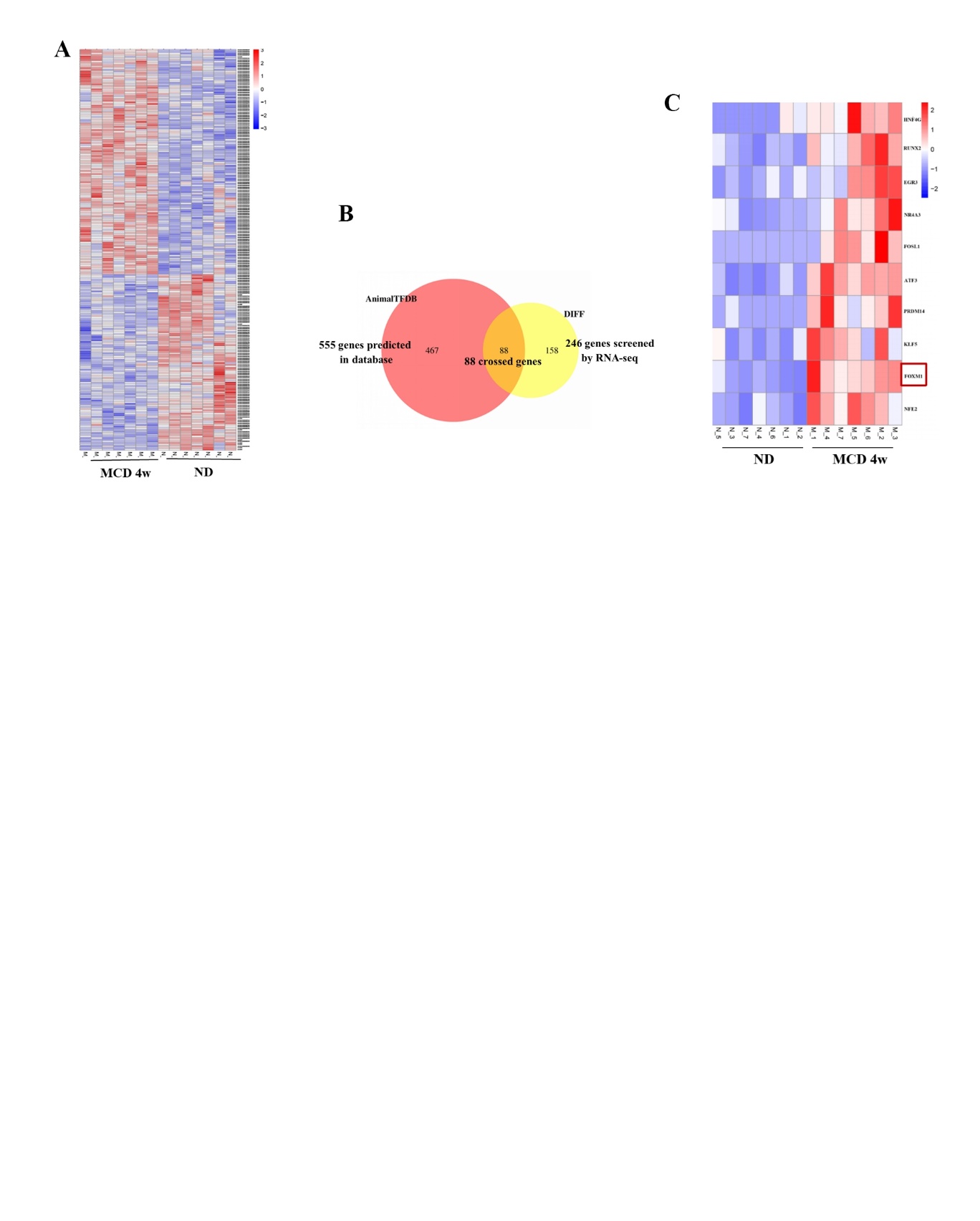


Supplementary Figure 3. **Screening of transcription factors for LAMP2A.** (A) Heatmap of differentially expressed transcription factors between mice fed a ND or MCD for 4 weeks identified via RNA sequencing. (B) Venn diagram showing the overlap of peaks between the transcription factors of LAMP2A identified via RNA sequencing and those predicted using a database. (C) Heatmap of the top 10 of the 88 overlapping transcription factors.


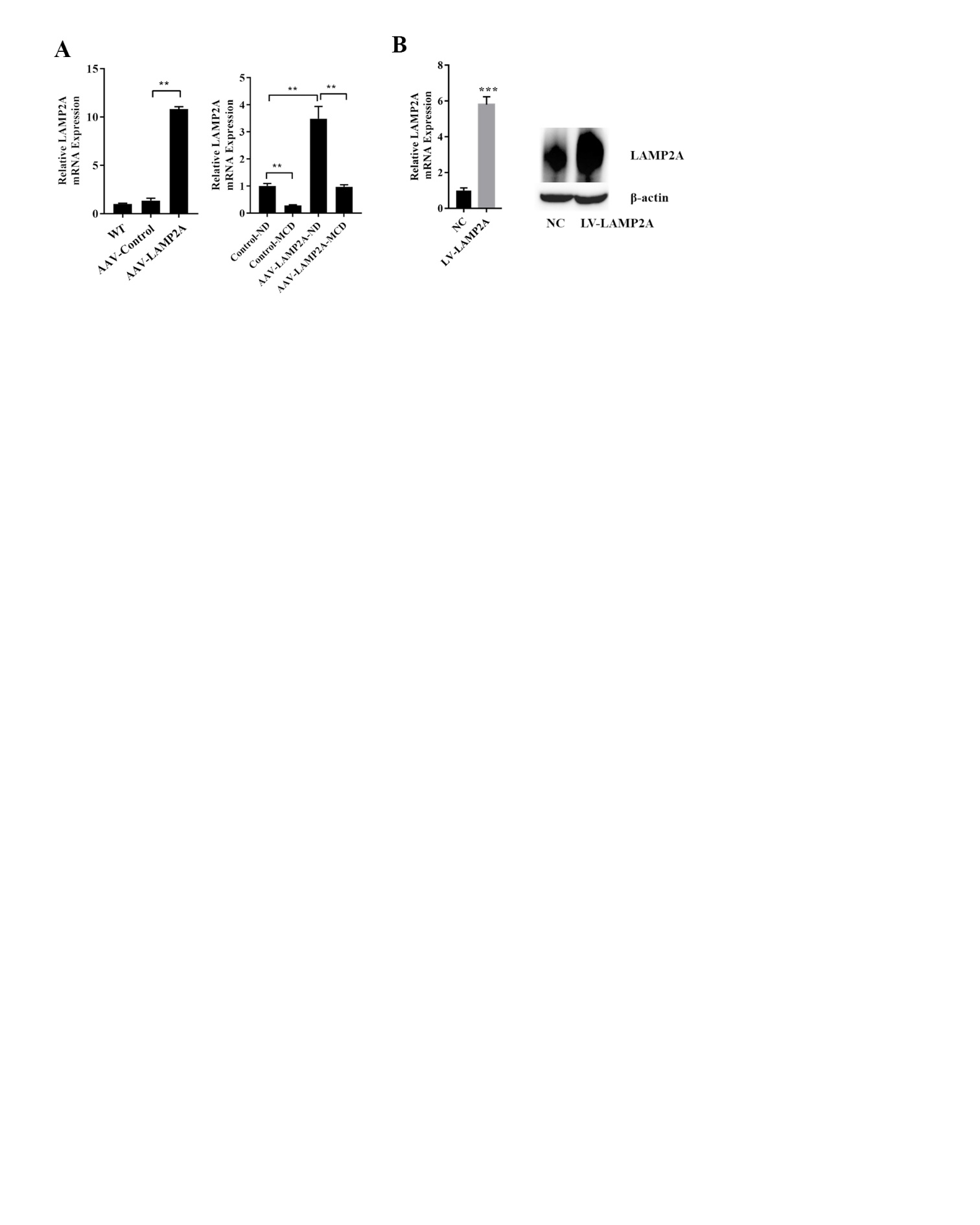


Supplementary Figure 4. **Successful induction of LAMP2A overexpression in the livers of mice (AAV-LAMP2A) and HepG2 cells (LV-LAMP2A).** (A) qRT‒PCR results for the mRNA expression of LAMP2A in liver tissues from control and AAV-LAMP2A mice (n = 3). (B) qRT‒PCR results for the mRNA expression of LAMP2A and western blot results for the protein expression of LAMP2A in negative control (NC) and LV-LAMP2A cells. All the data are expressed as means ± SDs (*, *P* < 0.05; **, *P* < 0.01; ***, *P* < 0.001). Significance was determined by two-tailed unpaired Student’s t test as appropriate with Prism software (GraphPad).


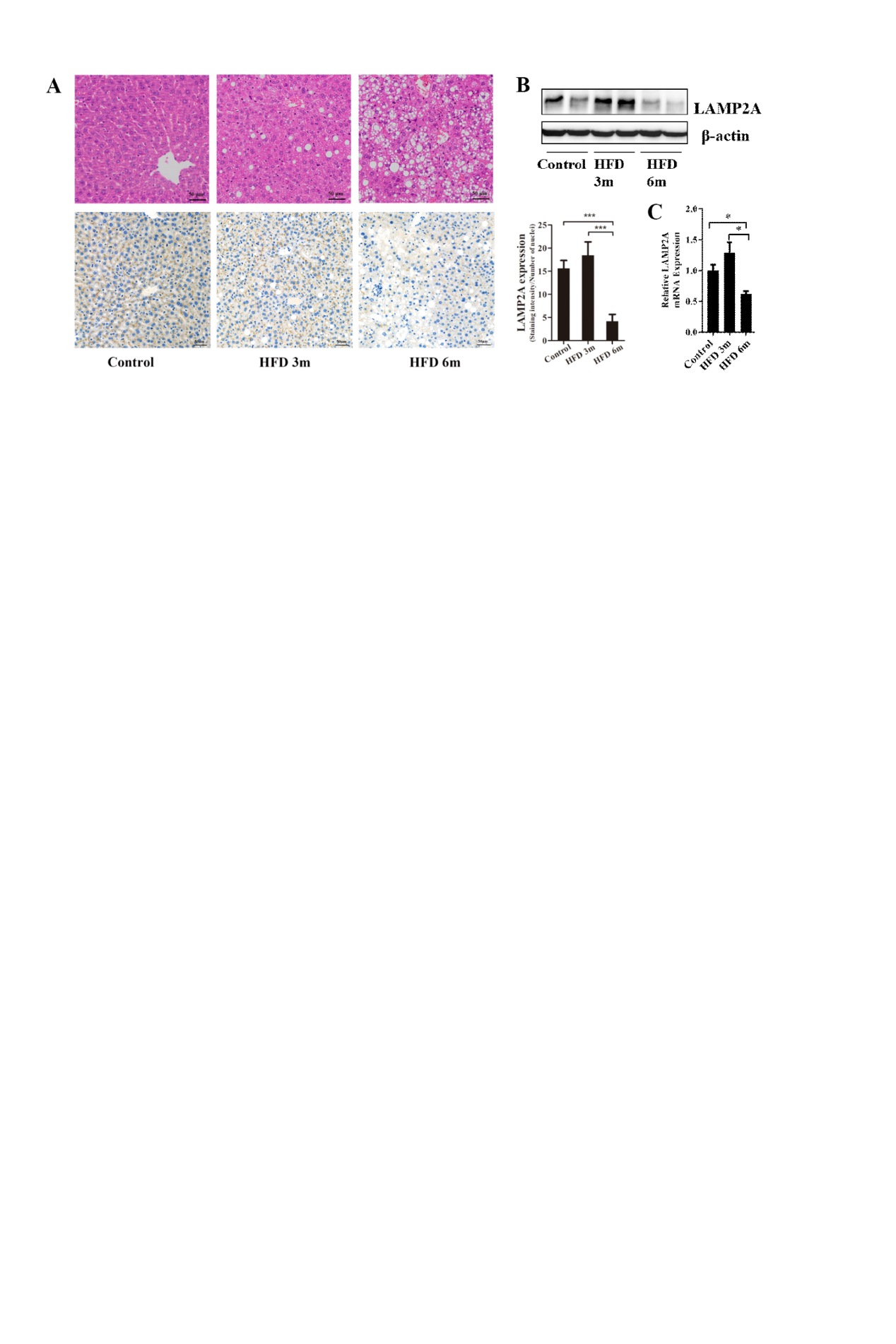


Supplementary Figure 5. **CMA is impaired during the progression of NAFLD to NASH in HFD-fed mice.** (A) Representative images of H&E staining and immunohistochemical staining for LAMP2A in liver tissues from HFD-fed mice and quantitative analysis of immunohistochemical staining using the Image-Pro Plus software (n = 5). (B) Representative western blot images of LAMP2A expression in liver tissues from HFD-fed mice. (C) qRT‒PCR results for the mRNA expression of LAMP2A in liver tissues from HFD-fed mice (n = 3). All the data are expressed as means ± SDs (*, *P* < 0.05; **, *P* < 0.01; **, *P* < 0.001). Significance was determined by two-tailed unpaired Student’s t test as appropriate with Prism software (GraphPad).

**Table 1. Primer sequences used for quantitative PCR**

| **Gene** | **Forward primer** | **Reverse primer** |
| --- | --- | --- |
| **Human** | | |
| *β-actin* | CGGTTCCGATGCCCTGAGGCTCTT | CGTCACACTTCATGATGGAATTGA |
| *LAMP2A* | GTGCAACAAAGAGCAGACTGT | GGCACAAGGAAGTTGTCGTC |
| *FOXM1* | AAACGGGAGACCTGTGATGG | ACCTTAACCTGTCGCTGCTC |
| *IL-6* | GTCAGGGGTGGTTTATTGCA | AGTGAGGAACAAGCCAGAGC |
| *TNF-α* | AACCTCCTCTCTGCCATCAA | GGAAGACCCCTCCCAGATAG |
| *IL-1β* | AGCAGAAAACATGCCCGTCT | GCAGTTGGGCATTGGTGTAG |
| *CD86* | GCTGTAACAGGGACTAGCACA | ACTGAAGTTAGCAGAGAGCAGG |
| *iNOS* | ACATGGCTCAACAGCCTGAA | CAAACACCAAGGTCATGCGG |
| *COL 1a1* | GAGGGCCAAGACGAAGACATC | CAGATCACGTCATCGCACAAC |
| *α-SMA* | CTATGAGGGCTATGCCTTGCC | GCTCAGCAGTAGTAACGAAGGA |
| *TGF-β* | TCCTGGCGATACCTCAGCAA | ACATGGGCTACAGGCTTGTCACT |
| *CHOP* | GCTCAGGAGGAAGAGGAGGA | TCCTGCTTGAGCCGTTCATT |
| *ATF4* | GAAGCGATTTAACGAGCGCC | ATCTTGGTTCCTGCCACGTT |
| *BIP* | CACTCCTGAAGGGGAACGTC | ACCACCTTGAACGGCAAGAA |
| *ATF6* | CAGCAGGAACTCAGGGAGTG | TGTTATGGGTGGTAGCTGGT |
| *XBP1* | CTGAGTCCGCAGCAGGTG | CTCTGGGGAAGGGCATTTGA |
| **Mouse** | | |
| *LAMP2A* | AGGTGCTTTCTGTGTCTAGAGCGT | AGAATAAGTACTCCTCCCAGAGCTGC |
| *LAMP2* | TTCAACACCCACTCCAACTC | AGCTGAGCCATTAGCCAAATA |
| *LAMP2B* | ATGTGCTGCTGACTCTGACCTCAA | TGGAAGCACGAGACTGGCTTGATT |
| *LAMP2C* | GGTGCTGGTCTTTCAGGCTTGATT | ACCACCCAATCTAAGAGCAGGACT |
| *FOXM1* | TTACTGCCCTTTCCTCGCAG | TCCCTGGCATGATTGGGAAC |
| *Il-6* | CCACTTCACAAGTCGGAGGCTTA | GCAAGTGCATCATCGTTGTTCATAC |
| *Tnf-α* | GACCCTCACACTCAGATCATCT | CCTCCACTTGGTGGTTTGCT |
| *F4/80* | GGAAAGCACCATGTTAGCTGC | CCTCTGGCTGCCAAGTTAATG |
| *Keratin8* | GAGGAGAGCAGGCTGGAGTC | GCTTCCCATCTCGGGTTTCA |
| *Keratin18* | CCTCAATCTGCTGAGACCAGTA | CTGTGGAGAGCGACATCCAT |
| *Nlrp3* | ATTACCCGCCCGAGAAAGG | CATGAGTGTGGCTAGATCCAAG |
| *Ccl2* | TTAAAAACCTGGATCGGAACCAA | GCATTAGCTTCAGATTTACGGGT |
| *Col 1a1* | TCTAGACATGTTCAGCTTTGTGGAC | TCTGTACGCAGGTGATTGGTG |
| *Col 1a2* | GGTGAGCCTGGTCAAACGG | ACTGTGTCCTTTCACGCCTTT |
| *Col 1a3* | TAGGACTGACCAAGGTGGCT | GGAACCTGGTTTCTTCTCACC |
| *α-SMA* | CGAGCCGAGAGTAGCAGTTGTAG | AGCCATTGTCGCACACGAG |
| *Ly6g* | TGCGTTGCTCTGGAGATAGA | CAGAGTAGTGGGGCAGATGG |
| *Chop* | TGCCTTTCACCTTGGAGACG | TCTGTCAGCCAAGCTAGGGA |
| *Atf4* | CCGGAAATTCGTCAACGAGC | ACTGCTGCTGGATTTCGTGA |
| *Bip* | TGTGTGTGAGACCAGAACCG | TAGGTGGTCCCCAAGTCGAT |
| *Atf6* | CTTCGGCAAGCAGAGACTTG | CAGAACCAACACCAGGCGA |
| *Xbp1* | TCCCTGACCATCACATTGCC | CAAATGACCCTCCCTGCGAT |
| *β-actin* | CAGCACAATGAAGATCAAGATC | CGGACTCATCGTACTCCTGCTT |
